# Classification of Hen Eggs by HPLC-UV Fingerprinting and Chemometric Methods

**DOI:** 10.3390/foods8080310

**Published:** 2019-08-01

**Authors:** Guillem Campmajó, Laura Cayero, Javier Saurina, Oscar Núñez

**Affiliations:** 1 Department of Chemical Engineering and Analytical Chemistry, University of Barcelona, Martí i Franquès 1-11, E08028 Barcelona, Spain; 2 Research Institute in Food Nutrition and Food Safety, University of Barcelona, Recinte Torribera, Av. Prat de la Riba 171, Edifici de Recerca (Gaudí), Santa Coloma de Gramenet, E08921 Barcelona, Spain; 3 Serra Húnter Fellow, Generalitat de Catalunya, Rambla de Catalunya 19-21, E08007 Barcelona, Spain

**Keywords:** HPLC-UV, fingerprinting, food classification, hen eggs, principal component analysis, partial least square-discriminant analysis

## Abstract

Hen eggs are classified into four groups according to their production method: Organic, free-range, barn, or caged. It is known that a fraudulent practice is the misrepresentation of a high-quality egg with a lower one. In this work, high-performance liquid chromatography with ultraviolet detection (HPLC-UV) fingerprints were proposed as a source of potential chemical descriptors to achieve the classification of hen eggs according to their labelled type. A reversed-phase separation was optimized to obtain discriminant enough chromatographic fingerprints, which were subsequently processed by means of principal component analysis (PCA) and partial least squares-discriminant analysis (PLS-DA). Particular trends were observed for organic and caged hen eggs by PCA and, as expected, these groupings were improved by PLS-DA. The applicability of the method to distinguish egg manufacturer and size was also studied by PLS-DA, observing variations in the HPLC-UV fingerprints in both cases. Moreover, the classification of higher class eggs, in front of any other with one lower, and hence cheaper, was studied by building paired PLS-DA models, reaching a classification rate of at least 82.6% (100% for organic vs. non-organic hen eggs) and demonstrating the suitability of the proposed method.

## 1. Introduction

In the last years, the interest of society in the food they purchase and consume has been raised. In this line, products with value-added due to specific particularities such as organic production, protected designation of origin (PDO), protected geographical indication (PGI), or those with fair-trade certification, are now receiving special attention. These labels not only ensure and guarantee food quality and traceability, but also mean an increment in its price in comparison with conventional products. 

Hen eggs are among the most commonly eaten foods worldwide, as they have a high nutritional value, cheap costs, and are widely employed in international cuisines. They consist of two parts: The egg white, which mainly consists of 85% water and 10% proteins (ovalbumin being the most abundant one) approximately, and the egg yolk, which is composed of almost 22% lipids [1,2]. Moreover, their intake provides all the essential amino acids, many vitamins (vitamin A, riboflavin, choline, vitamin B_12_, and vitamin B_9_), and minerals (phosphorus, potassium, iron, and zinc). 

In Europe, where almost 8 million tons of hen eggs were produced in 2017 [3], rules on their trade regarding production, hygiene, labelling, and marketing are laid down by the European Union (EU) [4,5,6]. Thereby, according to the European labelling eggs rule, each A quality category egg, which are those destined for human consumption, has to contain an identifier number code in its shell. Among other information that can be found on it, such as the country of origin (two-letter ISO - International Organization for Standardization- abbreviation code), the province, the municipality, and the producer establishment, the kind of hens and the breeding method employed are indicated by the first number digit:

Digit 0 is related to organic eggs (O), which means that they come from authorized and certified organic production farms. Thus, hens are fed with grown pasture and organic farming products, without employing transgenic substances nor antibiotics. The animals have a minimum space of 4 and 6 hens/m^2^ outdoors and indoors, respectively. These are the most expensive eggs. 

Digit 1 corresponds to free-range hen eggs (FR). In this case, their diet is mainly based on prepared cereal pellets, although grass can also be eaten. Antibiotics are mixed with food if needed. Moreover, similar space conditions to organic eggs are established. 

Digit 2 indicates barn hen eggs (B). Hens do not have outdoor access, as they live in densely populated vessels and therefore, their diet consists of the prepared pellets and there is no entrance of natural light. Further, antibiotics are systematically provided with feed. 

Digit 3 for eggs from caged hens (C), which are the cheapest ones. In these cages, hens can barely move (the minimum space allowed is of 12 hens/m^2^) and there is no access to natural light either. Medical additives are provided with feed. 

Due to the huge amount of produced eggs, two different frauds can be practiced. On one hand, in accordance with the European legislation, hen eggs have to reach the consumers within the 21 days of being laid [7], and their expiration date has to be fixed not more than 28 days after laying [6]. As there is no way to confirm whether those that are for sale are within the stipulated periods, some producers label them with erroneous dates, therefore giving a longer time before reaching their expiration date [8]. On the other hand, it is also difficult to distinguish hen eggs regarding their type. Although organic bodies may ensure the compliance of the established regulations, due to the high cost of the evaluation systems, some producers and distributors regulate themselves without adopting any national certification standard, leaving then the opportunity for food fraud [9]. 

The egg price increase from category 3 to 0 makes them susceptible to fraud, since a low category egg could be labelled as a superior one. Several methodologies have been previously developed in order to address egg authentication. For instance, profiling fatty acid composition by gas chromatography (GC), fitted with flame ionization detector (FID), in combination with chemometric techniques was proposed for the verification of organic against conventional eggs [1,10]. However, relatively time-consuming methodologies are usually required in order to determine the total lipid and fatty acid composition from the samples, also involving derivatization steps before GC separation. In another study, the carotenoid profile acquired by high-performance liquid chromatography and ultraviolet detection (HPLC-UV) was performed to classify both organic and conventional eggs [11]. Besides, in some cases, the authenticity of organic eggs and the assurance on their origin, was also approached by evaluating the level of several elements, including rare earth elements [12,13,14]. 

As can be seen, most of the methods described in the literature for egg authentication are based on targeted profiling approaches, which are focused on the specific determination of a given group of known selected chemicals. However, up to now, no specific biomarkers have been found in order to address hen eggs classification regarding their labelled class. Since many factors will affect the chemical composition of these products, non-targeted fingerprinting strategies that involve the determination of non-selective signals related to a range of potential discriminating compounds (i.e., spectrum or chromatogram), are promising approaches to address food authenticity issues [15,16,17,18,19]. As an example, a spectroscopic technique such as near infrared (NIR), in combination with principal component analysis (PCA), was proposed to achieve the classification of different type of eggs found in Chinese markets [20]. In the present work, HPLC-UV fingerprints recorded at 250 nm were proposed as a source of discriminant signals for hen eggs classification according to their production method by PCA and partial least squares-discriminant analysis (PLS-DA). 

## 2. Materials and Methods 

### 2.1. Chemicals and Standard Solutions

All the employed chemicals were of analytical grade. In the sample treatment, the acetonitrile and water (LC-MS Chromasolv^®^ quality) used were purchased from Sigma-Aldrich (St. Louis, MO, USA). Methanol (UHPLC-gradient grade) was obtained from PanReac AppliChem (Barcelona, Spain) and formic acid (≥98%) from Sigma-Aldrich. For the mobile phase, water was purified using an Elix 3 coupled to a Milli-Q system from Millipore Corporation (Burlington, MA, USA) and filtered through a 0.22 µm nylon membrane integrated into the Milli-Q system.

### 2.2. Instrumentation

An Agilent 1100 Series HPLC instrument equipped with a quaternary pump (G1311A), a degasser (G1322A), an autosampler (G1329A), a diode array detector (G1315B), and a PC with the Agilent Chemstation software, all of them from Agilent Technologies (Waldbronn, Germany), was employed. HPLC-UV fingerprints were obtained by reversed-phase mode using a Kinetex C18 porous-shell column (100 mm × 4.6 mm I.D., 2.6 µm particle size) from Phenomenex (Torrance, CA, USA) at room temperature. Chromatographic separation was performed under gradient elution mode, using 0.1% (*v/v*) formic acid aqueous solution (solvent A) and methanol (solvent B) as mobile phase components, following the next elution program: 0–20 min, linear gradient from 15% to 95% solvent B; 20–30 min, isocratic elution at 95% solvent B; 30–30.1 min, back to initial conditions; and from 30.1–35 min, at 15% solvent B for column re-equilibration. The mobile phase flow rate was 0.4 mL/min, and the injection volume was 5 µL. The HPLC-UV fingerprints were registered at 250 nm. 

### 2.3. Samples and Sample Treatment

Characterization and classification studies were carried out by analyzing 173 hen egg samples purchased from local markets (Barcelona, Spain). Table 1 classifies them according to their typology and manufacturer and defines their specified size as well as the number of samples. 

Sample extraction was performed following a previously described method [21], with some modifications. Briefly, 0.3 g of homogenized egg sample were weighed in an Eppendorf tube (Deltalab, Rubí, Spain), mixed with 1 mL of an acetonitrile:water 80:20 (*v/v*) solution by stirring in a Vortex (Stuart, Stone, United Kingdom) for 30 s, and then, centrifuged (Allegra^TM^ 64R Centrifugue, Beckman Coulter, L’Hospitalet de Llobregat, Spain) for 10 min at 14,000 rpm and 4 °C. The supernatant extract was then filtered through 0.22 µm filter (Scharlab, Sentmenat, Spain) and stored at −18 °C in 2 mL glass injection vials until HPLC-UV analysis.

Moreover, a quality control (QC), which aimed to evaluate the repeatability of the method and the robustness of the chemometric results, was prepared by mixing 50 µL of each sample extract. A QC and a blank of acetonitrile were injected every 10 randomly injected samples. 

### 2.4. Data Analysis

PCA and PLS-DA calculations were made by using SOLO 8.6 chemometric software (Eigenvector Research [22], Manson, WA, USA). Theoretical background of these methods in a detailed way is addressed elsewhere [23].

X-data matrices in both PCA and PLS-DA analysis consisted of the HPLC-UV chromatographic fingerprints obtained at 250 nm (absorbance intensities vs. retention time), whereas the Y-data matrix in PLS-DA defined each sample class. In order to improve the data quality, HPLC-UV chromatograms were smoothed, baseline-corrected, aligned, and autoscaled. Scatter plots of scores from principal components (PCs), in PCA, and latent variables (LVs), in PLS-DA, were used to study the distribution of samples, revealing patterns that could be correlated to their characteristics. In order to build both PCA and PLS-DA models, the first significant minimum point of the cross validation (CV) error from a Venetian blind approach was considered to be the most appropriate number of PCs or LVs, respectively. 

## 3. Results and Discussion

### 3.1. HPLC-UV Chromatographic Separation

**Figure 1:**
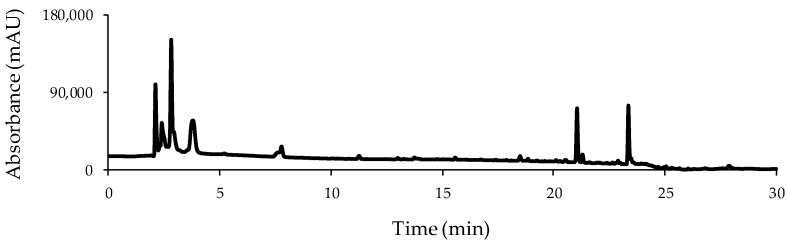
High performance liquid chromatography with ultraviolet detection (HPLC-UV) chromatogram at 250 nm obtained for a selected barn hen egg sample under the proposed gradient elution program (Section 2.2).

This work aimed to develop a HPLC-UV fingerprinting approach for the classification and discrimination of hen eggs according to their labelled typology. Thus, in order to obtain the richest chromatographic fingerprints, after a slightly modified simple liquid–liquid extraction procedure [19], the obtained extract of a B egg sample was employed for the optimization of the chromatographic separation by reversed-phase mode using 0.1% aqueous formic acid and methanol as mobile phase components. In a first separation consisting of a universal gradient, where methanol increased from 10% to 90% in 30 min, several compounds with different peak intensities were detected, although most of them elute close to the column dead volume. Thereby, different initial methanol percentages as well as the combination of gradient and isocratic steps were tested. As a compromise between the number of detected peaks, resolution, and analysis time, a final gradient consisting of an increase of methanol from 15% to 95% in 20 min followed by an isocratic step at 95% methanol for 10 min was selected. Figure 1 shows the obtained HPLC-UV chromatographic fingerprint registered at 250 nm for a B egg sample with the proposed gradient program. 

### 3.2. HPLC-UV Fingerprints

**Figure 2:**
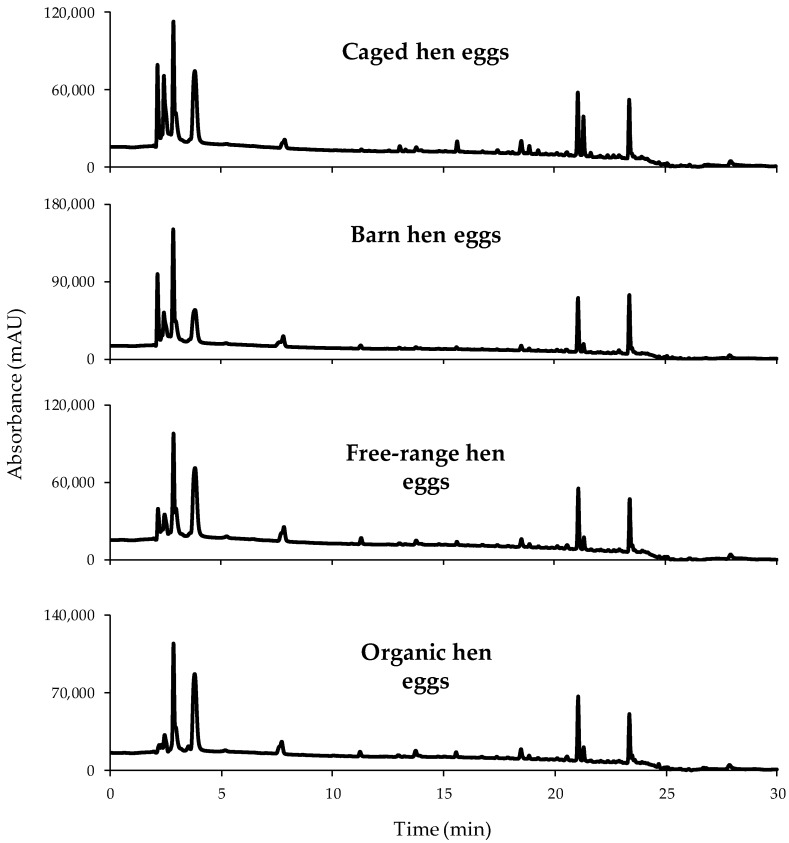
High performance liquid chromatography with ultraviolet detection (HPLC-UV) chromatographic fingerprints acquired at 250 nm for a selected sample within each egg type.

A total of 173 egg samples were analyzed by the proposed HPLC-UV method for classification purposes. For instance, Figure 2 shows the chromatograms at 250 nm for each one of the egg sample groups (O, FR, B, and C) analyzed. At a first glance, similar chromatographic fingerprints were obtained independently of the egg type. In fact, according to the retention times, the detected compounds seemed to be the same in each of them. However, variations associated to peak intensities, as well as their abundance within the different peak signals detected in a same sample, can be easily remarked. Therefore, these chromatographic fingerprints were evaluated and proposed as chemical descriptors to achieve sample classification.

### 3.3. Classification of Samples According to Egg Type: PCA Study

**Figure 3:**
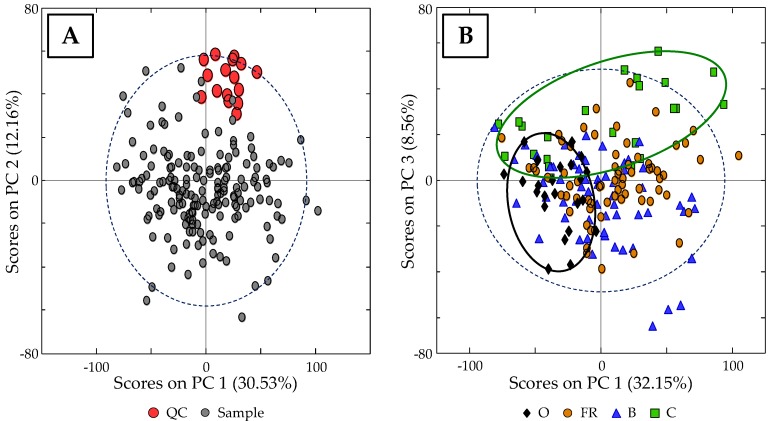
(**A**) Scores plot of PC1 vs. PC2 when using HPLC-UV fingerprints registered at 250 nm as chemical descriptors, showing a correct behavior of quality control (QC) samples. (**B**) Scores plot of PC1 vs. PC3, without including QC samples, showing a slight trend of organic hen (O) and caged hen (C) eggs.

As a first approach, a non-supervised PCA study was performed to evaluate the usefulness of HPLC-UV fingerprints for eggs classification according to their type. For that purpose, a data matrix (189 × 4506, samples × variables), which consisted of the recorded absorbance signals at 250 nm as a function of time for the analyzed egg samples and the QCs, was built. Moreover, data were pretreated as mentioned in Section 2.4, not only to reduce noise interferences, peak shifting, and baseline drifts, but also to provide the same weight to each variable by suppressing differences in their magnitude and amplitude scales. As a first result, the plot of scores of PC1 vs. PC2 (seven PCs were chosen for the PCA analysis), which is displayed in Figure 3A, shows that QC samples form a clear group (without any trend associated to a systematic error) in the upper side of the diagram, allowing the consideration of the obtained chemometric results. As can be seen in the plot of scores PC1 vs. PC3 shown in Figure 3B (seven PCs were also chosen for the supervised analysis), when excluding QC samples, even though there is not an evident discrimination among the samples, both the highest (O eggs) and lowest (C eggs) quality eggs predominate above and on the left of the plot, respectively. Up to this point, the proposed HPLC-UV fingerprints appeared to be adequate chemical descriptors at least for the distinction of O and C eggs, although PCA is only a non-supervised exploratory chemometric method. Therefore, in order to better exploit the obtained data and to improve the results on sample distribution, a supervised PLS-DA chemometric classification method was evaluated. 

### 3.4. Classification of Samples According to Egg Type: PLS-DA Study

**Figure 4:**
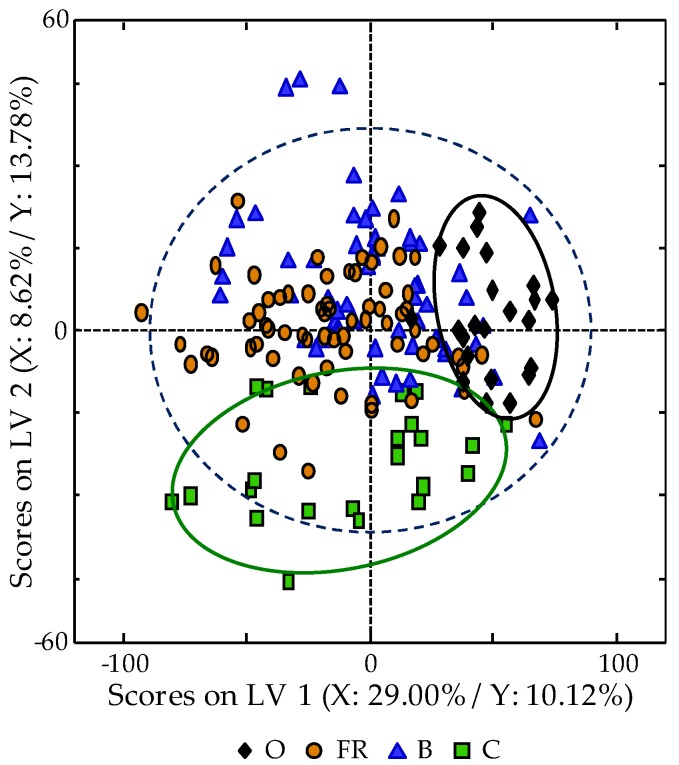
Partial least square-discriminant analysis (PLS-DA) scores plots of LV1 vs. LV2 when using HPLC-UV fingerprints registered at 250 nm as chemical descriptors.

The supervised chemometric study of all the analyzed egg samples was carried out by PLS-DA. For this reason, in addition to the X-data matrix previously described in the PCA study, a Y-matrix indicating the membership of each sample (O, FR, B, and C eggs) was used. The obtained scores plot of LV1 vs. LV2 (six LVs were chosen as optimum for the PLS-DA model, as detailed in Section 2.4), which is shown in Figure 4, improves non-supervised chemometric results as expected, and the obtained distribution seem to be directly related to the hens breeding method employed. In fact, eggs of hens with organic diet (O eggs) follow a particular trend mainly due to LV1, whereas LV2 affects those obtained from hens fed with a cereal-based diet and reared in cages (C eggs). On the other hand, in between these two group samples, both FR and B eggs, which as C eggs, are also collected from hens with a cereal-based diet but with better breeding conditions, apparently appeared randomly distributed. 

**Figure 5:**
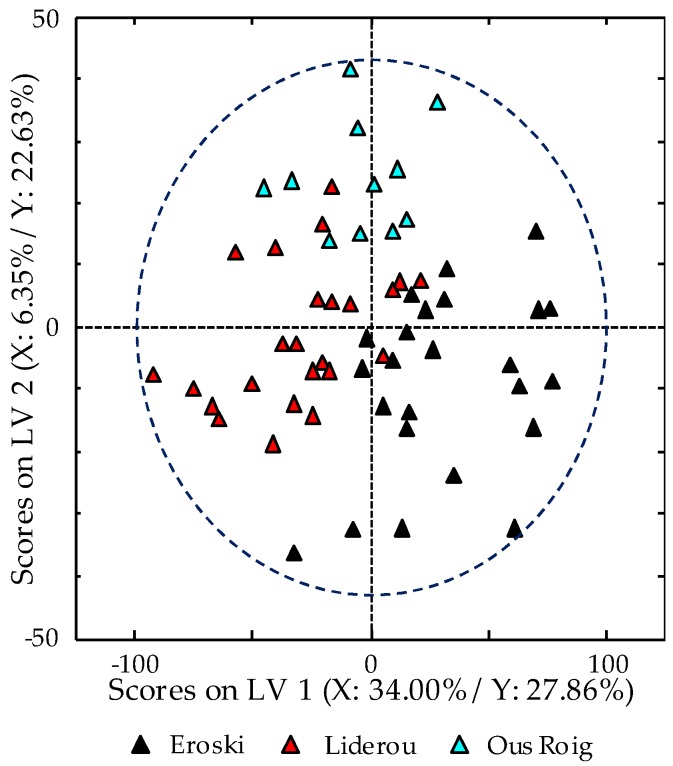
PLS-DA scores plots of LV1 vs. LV2 for B hen egg when using HPLC-UV chromatographic fingerprints registered at 250 nm as chemical descriptors.

A fact that should be taken into consideration is the number of manufacturers involved within the employed samples for each typology. While for O and C eggs all samples belonged only to one manufacturer, FR and B groups came from three. Thus, although according to the EU, same rules on the breeding process are established for a given quality egg class, additional sources of variance, such as the cereals employed in hens diet or the available grass and plants, could suppose a differential factor. Therefore, the applicability of HPLC-UV fingerprints as chemical descriptors to distinguish between the egg manufacturers was also evaluated by means of PLS-DA. For instance, Figure 5 shows the obtained scores plot of LV1 vs. LV2 when a 4 LVs PLS-DA model was built only for B egg samples. As can be observed, B eggs are clearly grouped according to their manufacturer, and thus, the proposed chromatographic fingerprints seem to be capable to remark these differences between different origins of production. 

Moreover, the size of the studied eggs was also evaluated by this methodology, as reported in Figure S1 (Supplementary Material). For that purpose, a matrix containing B and C egg samples, which were the only available classes labelled by size, was constructed. A clear distinction between M and L size eggs was achieved, independently of their class (B or C), denoting changes in the phytochemical fingerprint related to this morphological characteristic. 

### 3.5. Supervised PLS-DA Method Validation

As the main goal of the present work was the discrimination of hen eggs according to their labelled class, and in order to demonstrate the applicability of the proposed method, the classification of higher class eggs in front of any other with one lower, and hence cheaper, was studied by building paired PLS-DA models (i.e., O vs. FR, B and C eggs; FR vs. B and C eggs; and B vs. C eggs). As can be observed in Figure S2 (Supplementary Material), the number of LVs employed to generate each classificatory model was selected considering the first significant minimum point of the CV error average as the most appropriate one. 

**Figure 6:**
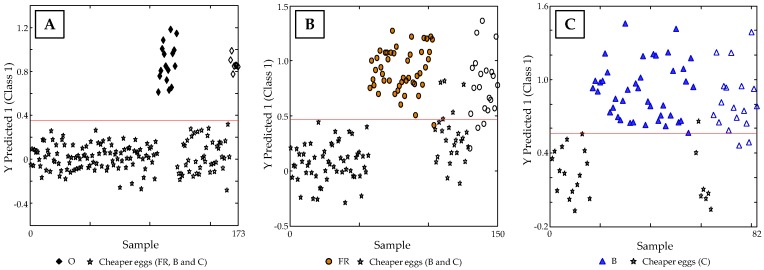
Sample vs. Y predicted 1 Scores plot for (**A**) O vs. FR, B and C eggs, (**B**) FR vs. B and C, and (**C**) B vs. C.

For predicting the egg classes, the chemometric model was established using 70% of samples of each group as calibration set, while the remaining 30% was employed as “unknown” set for validation purposes. As can be seen in Figure 6A, O eggs, which are the most expensive ones, were clearly discriminated from those with lower prizes, reaching a classification rate of 100%. Further, while for the PLS-DA model of FR in front of B and C eggs (Figure 6B) a discrimination capacity of 82.6% was accomplished, B in front of C eggs (Figure 6C) resulted to be of 88%. 

## 4. Conclusions

In this work, HPLC-UV chromatograms acquired at 250 nm have proved to be useful discriminant fingerprints for the classification of hen eggs according to their labelled typology. The PLS-DA models built for each egg category in front of those with lower one have reached at least a classification rate of 82.6%, showing satisfactory results of prediction. The distinction among organic and non-organic eggs has been especially satisfactory, in which 100% of sensitivity and selectivity has been reached. Moreover, the chromatographic fingerprints have also shown differences in egg phytochemical content among samples with different size independently of their type, as well as different manufacturers between samples from the same class. 

Even though HPLC-UV fingerprints provided satisfactory results, the perfect classification of the four labelled hen egg groups was not achieved. At this point and in order to improve them, the evaluation of a new matrix, such as the egg yolk, rather than using the whole egg, could be an improvement to solve this problematic. Besides, fluorescence detection, which is characterized to be more selective than UV detection, could be proposed as an alternative detection technique for better descriptive models. 

Finally, compared with biomarker-based strategies, the principal advantage of fingerprinting approaches is that the identification and quantification of selective species of each class are not essential for a successful sample classification. Here, in this regard, despite that specific markers have not been found, subtle differences in the content of components up- or down-expressed among classes have been exploited as the basis of the classification models. However, future work should also be directed towards biomarkers identification in order to address hen eggs authentication. 

## Figures and Tables

**Table 1:** Description of the egg samples analyzed.

Egg Type	Manufacturer	Egg Size	Number of Samples
Organic hen eggs (O)	ViuBi	M/L	23
Free-range hen eggs (FR)	Vall de Mestral	-	23
	Ous Roig (Ebre)	-	23
	Ous Roig	L/XL	22
Barn hen eggs (B)	Liderou	M	24
	Eroski	L	24
	Ous Roig	L	11
Caged hen eggs (C)	Eroski	M	12
	Eroski	L	11

## Supplementary Materials

The following are available online at https://www.mdpi.com/2304-8158/8/8/310/s1, Figure S1: PLS-DA scores plots of LV1 vs. LV2 for M and L size eggs when using HPLC-UV chromatographic fingerprints registered at 250 nm as chemical descriptors. Figure S2: Latent variable number vs. CV classification error average plots for the built PLS-DA models of: (A) O vs. FR, B and C eggs, (B) FR vs. B and C, and (C) B vs. C.
