# Supplementary material for: Classification of Hen Eggs by HPLC-UV Fingerprinting and Chemometric Methods
Source: Foods. 2019 Aug 1;8(8):310. doi: 10.3390/foods8080310 (PMC6723454; doi:10.3390/foods8080310)
Supplement: Supplementary file 1 [file foods-08-00310-s001.pdf]

foods

Article

Classification of Hen Eggs by HPLC-UV Fingerprinting and Chemometric Methods

Guillem Campmajó [1], Laura Cayero [1], Javier Saurina [1,2] and Oscar Núñez [1,2,3,*]

[1] Department of Chemical Engineering and Analytical Chemistry, University of Barcelona, Martí i Franquès 1-11, E08028 Barcelona, Spain; campma03@gmail.com (G.C.); laura.cayero@gmail.com (L.C.); nereant7@gmail.com (N.N.), xavi.saurina@ub.edu (J.S.)
[2] Research Institute in Food Nutrition and Food Safety, University of Barcelona, Recinte Torribera, Av. Prat de la Riba 171, Edifici de Recerca (Gaudí), Santa Coloma de Gramenet, E08921 Barcelona, Spain
[3] Serra Húnter Fellow, Generalitat de Catalunya, Rambla de Catalunya 19-21, E08007 Barcelona, Spain
* Correspondence: oscar.nunez@ub.edu; Tel.: +34-93-403-3706

Received: date; Accepted: date; Published: date

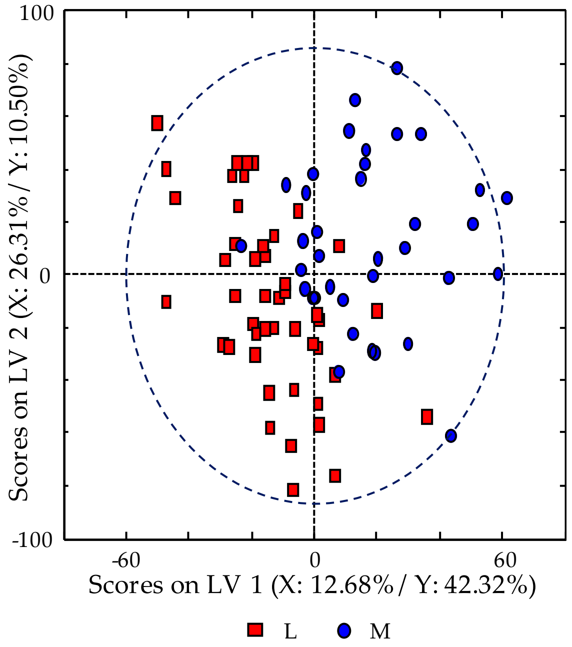

Figure S1. PLS-DA scores plots of LV1 *vs* LV2 for M and L size eggs when using HPLC-UV chromatographic fingerprints registered at 250 nm as chemical descriptors.

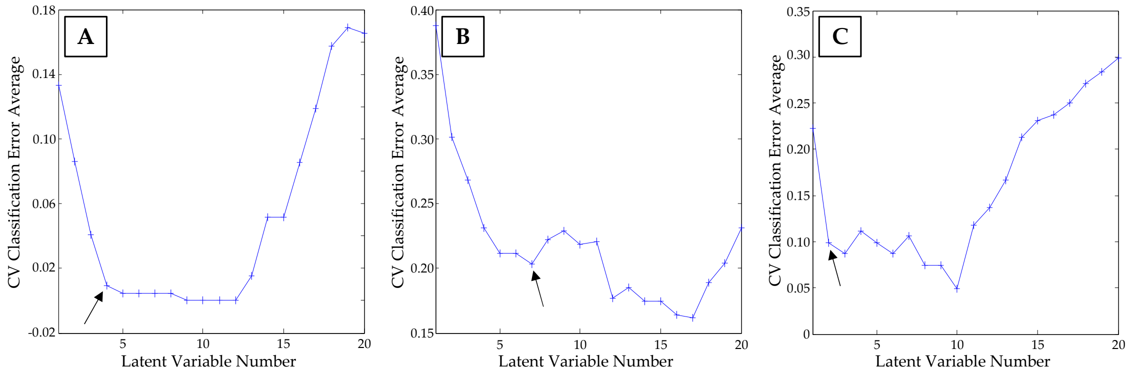

Figure S2. Latent variable number *vs* CV classification error average plots for the built PLS-DA models of: (**A**) O *vs* FR, B and C eggs, (**B**) FR *vs* B and C, and (**C**) B *vs* C.
